# Ablation of *Mea6/cTAGE5* in oligodendrocytes significantly impairs white matter structure and lipid content

**DOI:** 10.1093/lifemeta/load010

**Published:** 2023-03-23

**Authors:** Tiantian Ma, Wei Mao, Shaohua Zhang, Yaqing Wang, Tao Wang, Jinghua Liu, Lei Shi, Xiang Yu, Rong Xue, Guanghou Shui, Zhiheng Xu

**Affiliations:** State Key Laboratory of Molecular Developmental Biology, Institute of Genetics and Developmental Biology, Chinese Academy of Sciences, Beijing 100101, China; University of Chinese Academy of Sciences, Beijing 100049, China; State Key Laboratory of Brain and Cognitive Science, Beijing MRI Center for Brain Research, Institute of Biophysics, Chinese Academy of Sciences, Beijing 100101, China; Sino-Danish College, University of Chinese Academy of Sciences, Beijing 100049, China; State Key Laboratory of Molecular Developmental Biology, Institute of Genetics and Developmental Biology, Chinese Academy of Sciences, Beijing 100101, China; University of Chinese Academy of Sciences, Beijing 100049, China; State Key Laboratory of Molecular Developmental Biology, Institute of Genetics and Developmental Biology, Chinese Academy of Sciences, Beijing 100101, China; University of Chinese Academy of Sciences, Beijing 100049, China; State Key Laboratory of Molecular Developmental Biology, Institute of Genetics and Developmental Biology, Chinese Academy of Sciences, Beijing 100101, China; Sino-Danish College, University of Chinese Academy of Sciences, Beijing 100049, China; State Key Laboratory of Molecular Developmental Biology, Institute of Genetics and Developmental Biology, Chinese Academy of Sciences, Beijing 100101, China; Sino-Danish College, University of Chinese Academy of Sciences, Beijing 100049, China; State Key Laboratory of Molecular Developmental Biology, Institute of Genetics and Developmental Biology, Chinese Academy of Sciences, Beijing 100101, China; School of Life Sciences, Peking University, Beijing 100871, China; State Key Laboratory of Brain and Cognitive Science, Beijing MRI Center for Brain Research, Institute of Biophysics, Chinese Academy of Sciences, Beijing 100101, China; Sino-Danish College, University of Chinese Academy of Sciences, Beijing 100049, China; Center for Excellence in Brain Science and Intelligence Technology, Chinese Academy of Sciences, Beijing 100101, China; Beijing Institute for Brain Disorders, Collaborative Innovation Center for Brain Disorders, Capital Medical University, Beijing 100069, China; State Key Laboratory of Molecular Developmental Biology, Institute of Genetics and Developmental Biology, Chinese Academy of Sciences, Beijing 100101, China; University of Chinese Academy of Sciences, Beijing 100049, China; State Key Laboratory of Molecular Developmental Biology, Institute of Genetics and Developmental Biology, Chinese Academy of Sciences, Beijing 100101, China; University of Chinese Academy of Sciences, Beijing 100049, China; Center for Excellence in Brain Science and Intelligence Technology, Chinese Academy of Sciences, Beijing 100101, China; Beijing Institute for Brain Disorders, Collaborative Innovation Center for Brain Disorders, Capital Medical University, Beijing 100069, China

**Keywords:** Mea6, oligodendrocyte, hypomyelination, myelin lipidomic analysis

## Abstract

Lipid-rich myelin is a special structure formed by oligodendrocytes wrapping neuronal axons. Abnormal myelin sheath is associated with many neurological diseases. Meningioma-expressed antigen 6 (Mea6)/cutaneous T cell lymphoma-associated antigen 5C (cTAGE5C) plays an important role in vesicle trafficking from the endoplasmic reticulum (ER) to Golgi, and conditional knockout (cKO) of *Mea6* in the brain significantly affects neural development and brain function. However, whether the impaired brain function involves the development of oligodendrocytes and white matter beyond neurons remains unclear. In this study, by using different models of diffusion magnetic resonance imaging, we showed that cKO of *Mea6* in oligodendrocytes leads to significant impairment of the gross and microstructure of the white matter, as well as a significant decrease of cholesterol and triglycerides in brains. Our lipidomic analysis of purified myelin sheath for the first time showed that *Mea6* elimination in oligodendrocytes significantly altered the lipid composition in myelin lipidome, especially the proportion of very long chain fatty acids (VLCFAs). In particular, the levels of most VLCFA-containing phosphatidylcholines were substantially lower in the myelin sheath of the cKO mice. The reduction of VLCFAs is likely due to the downregulated expression of *elongation of very long chain fatty acids* (*ELOVLs*). Our study of an animal model with white matter malformation and the comprehensive lipid profiling would provide clues for future studies of the formation of myelin sheath, myelin lipids, and the pathogenesis of white matter diseases.

## Introduction

In addition to gray matter (neuron-rich regions), an increase in white matter volume during development is also important for brain function [1, 2]. During development, white matter expands around the corpus callosum (CC) and embeds in the gray matter. More than half of adult human brain is made up of white matter [1]. White matter dysfunction is associated with a variety of neurological disorders, such as X-linked leukodystrophies, multiple sclerosis, Alzheimer’s disease, and schizophrenia [3–5].

White matter is predominantly composed of lipid-rich mature oligodendrocytes which spirally wrap neuronal axons [6]. After initiating myelination, oligodendrocytes rapidly expand their surface area and synthesize large amounts of lipids in a short period, forming a special tightly wrapped lipid bilayer structure, named myelin sheath [7]. Although cholesterol, phospholipids, and glycolipids are the most abundant lipid species in all biological membranes, the content of cholesterol in myelin (~40%) is much higher than in other membrane structures (~25%) while phospholipid is lower (~40% versus ~65%) [8]. Cholesterol inserted into the myelin bilayer increases viscosity, and cooperates with other lipids and proteins to improve myelin stability [8]. Although oligodendrocytes can take up lipids from surrounding cells to assist myelination, abnormal myelination due to blockage of lipid synthesis cannot be efficiently improved by lipids in the external environment, especially in adulthood and old age [9, 10].

In addition to the major types of lipids, lipids can be divided into common fatty acids (usually containing 12–22 carbon atoms) and very long chain (23 or more carbon atoms) fatty acids (VLCFAs) according to carbon chain length [11]. VLCFAs act as structural lipids and play important roles in maintaining liver homeostasis, retinal function, spermatogenesis, myelin maintenance, and skin barrier formation [12]. The rate-limiting step in VLCFA synthesis is catalyzed by enzymes, elongation of very long chain fatty acids (ELOVL1–ELOVL7). Each ELOVL has specificity for substrate selection [13]. Myelin sheath, especially sphingolipids, contains abundant VLCFAs. *Elovl1* mutations cause multiple neurological disorders including hypomyelination [14]. *Elovl1* deletion mice show severe hypomyelination in the CC and markedly shortened carbon chain length of sphingolipids, leading to poor motor coordination and reduced survival rate [14]. Many vacuoles adjacent to Olig2-positive cells were detected in the brain slices of patients with *ELOV4* mutation [15]. These studies suggest a potential role of VLCFAs in myelin sheath maintenance.

Accurate quantification of myelin content is important for clinical diagnosis. Several imaging techniques are used to quantify myelin sheath, including magnetic resonance imaging (MRI) [16]. Diffusion magnetic resonance imaging (dMRI) is an *in vivo* imaging technique to explore the microstructure of nervous tissue and neural connectome between different regions in the brain by detecting molecular water random movement. There are various diffusion models developed, including diffusion tensor image (DTI) [17], diffusion kurtosis image (DKI) [18, 19], white matter tract integrity (WMTI) [20], etc. DTI technique assumes that the water molecule’s diffusion displacement follows a Gaussian distribution function in the biological system. However, anisotropic tissues, such as the brain, have complex cellular structures. The molecules’ diffusion usually deviates from Gaussian distribution, which can be quantified by DKI. The WMTI model proposes two-exchanging compartments of white matter to quantify the diffusivity in intra-axonal space (IAS) and extra-axonal space (EAS), which can probe the microstructure of myelin sheaths more accurately.

Meningioma-expressed antigen 6 (Mea6), also known as cutaneous T cell lymphoma-associated antigen 5C (cTAGE5), plays a crucial role in the initial assembly of the COPII complex and cargo trafficking packaged by COPII vesicles in ER exit site (ERES) from the ER to the Golgi [21, 22]. *Mea6* is highly expressed in the pancreas, liver, and brain [22]. Specific abrogation of *Mea6/cTAGE5* in mouse pancreas caused blocked insulin secretion and glucose intolerance [23]. Conditional knockout (cKO) of *Mea6/cTAGE5* in the liver led to defects in very low density lipoprotein (VLDL) secretion and fatty liver [21], and elimination of *Mea6/cTAGE5* from the whole brain led to serious defects in brain development including thinner CC [22]. *Mea6/cTAGE5* was also shown to be important for neuron development and brain function [22, 24, 25].

Here, we show that mice with oligodendrocyte lineage-specific *Mea6* knockout (referred to cKO mice hereafter) display significantly reduced volume of white matter and the proportion of myelin weight to brain weight. We performed the first full lipidomic biochemical analyses of myelin and detected global changes in myelin lipid composition, especially the decrease of VLCFAs. Moreover, we found that *Mea6* KO may affect the elongation of VLCFAs via affecting the expression of *ELOVLs*.

## Results

### *Mea6/cTAGE5* cKO in oligodendrocytes results in thinning of CC and disturbed microstructure of white matter

**Figure 1:**
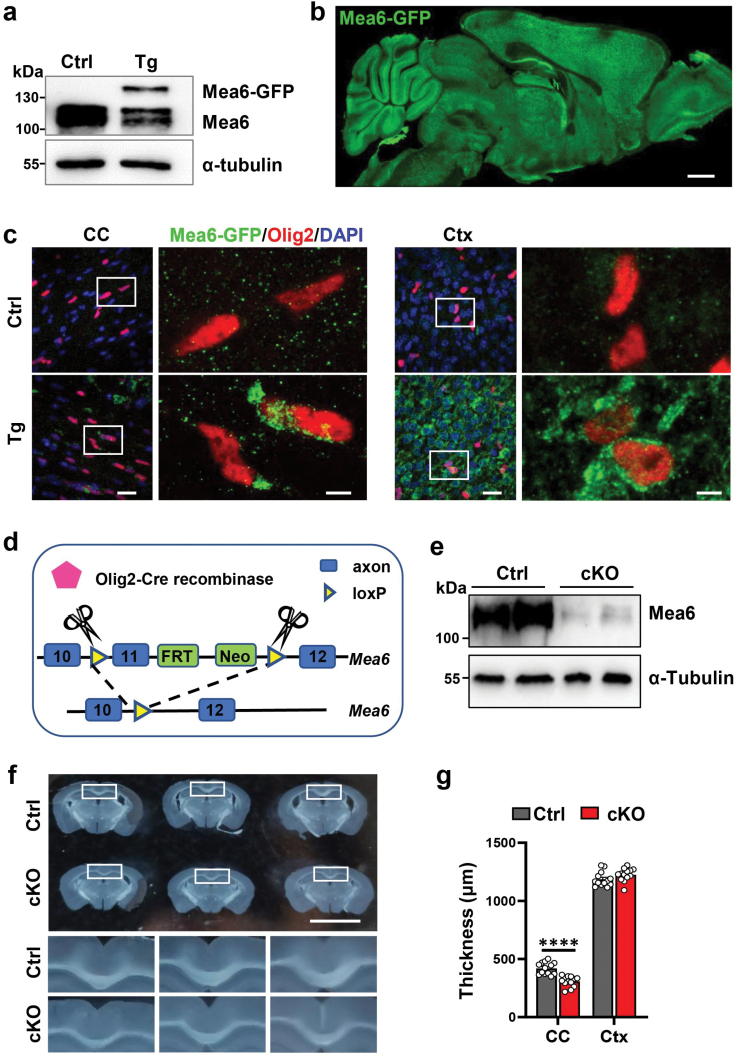
cKO of *Mea6* in oligodendrocyte results in thinning of CC. (a) Western blot verification of the fusion between EGFP and Mea6 in transgenic mice (Tg). (b) Immunostaining of the sagittal section of brain. Scale bar: 1 mm. (c) Olig2^+^ oligodendrocytes in the CC and cortex show EGFP signal in Tg mice, but not in Ctrl mice. The images in the white box are enlarged and displayed on the right. Ctx, cortex. The scale bars in the left and right panels are 20 μm and 5 μm, respectively. (d) Strategy of *Mea6* cKO in oligodendrocytes. (e) Immunoblotting of Mea6 in primary cultured oligodendrocytes. (f) Images of coronal brain slices of P160 mice. Inset images are magnified in the lower panels. Scale bars: 5 mm. (g) Quantification for the thickness of CC and Ctx in (f). Ctrl: *n* = 13/3, cKO: *n* = 13/3. All data are mean ± SEM. Data were analyzed via Student’s *t-*test. ^****^P < 0.0001.

Oligodendrocytes require the synthesis and transport of a large amount of lipid and protein during myelination. Ablation of *Mea6*/*cTAGE5* from the whole brain led to a significantly thinner CC due to defects in either neurons or oligodendrocytes [22]. We inspected the expression of *Mea6*/*cTAGE5* in oligodendrocytes, but found that the available Mea6 antibody was not suitable for paraformaldehyde (PFA) fixed tissues. We, therefore, knocked in EGFP coding sequence at the C-terminus of *Mea6* and confirmed their fused expression (Fig. 1a). GFP signal was detected throughout the brain in both neurons and Olig2-positive cells in the CC and cortex (Fig. 1b and c).

We went on to cross *Mea6*^*fl/+*^*;Olig2-Cre* mice with *Mea6*^*fl/fl*^ mice to generate the cKO mice (Fig. 1d; Supplementary Fig. S1a). Almost complete loss of *Mea6* expression was confirmed in cultured cKO oligodendrocytes (Fig. 1e), but not in neurons (Supplementary Fig. S1b). We inspected the brain slices from the 5-month-old cKO and control (Ctrl) mice and found that the CC in cKOs was more visibly transparent (Fig. 1f) and much thinner (307.1 ± 45.7 μm vs 419.6 ± 51.9 μm) (Fig. 1g).

**Figure 2:**
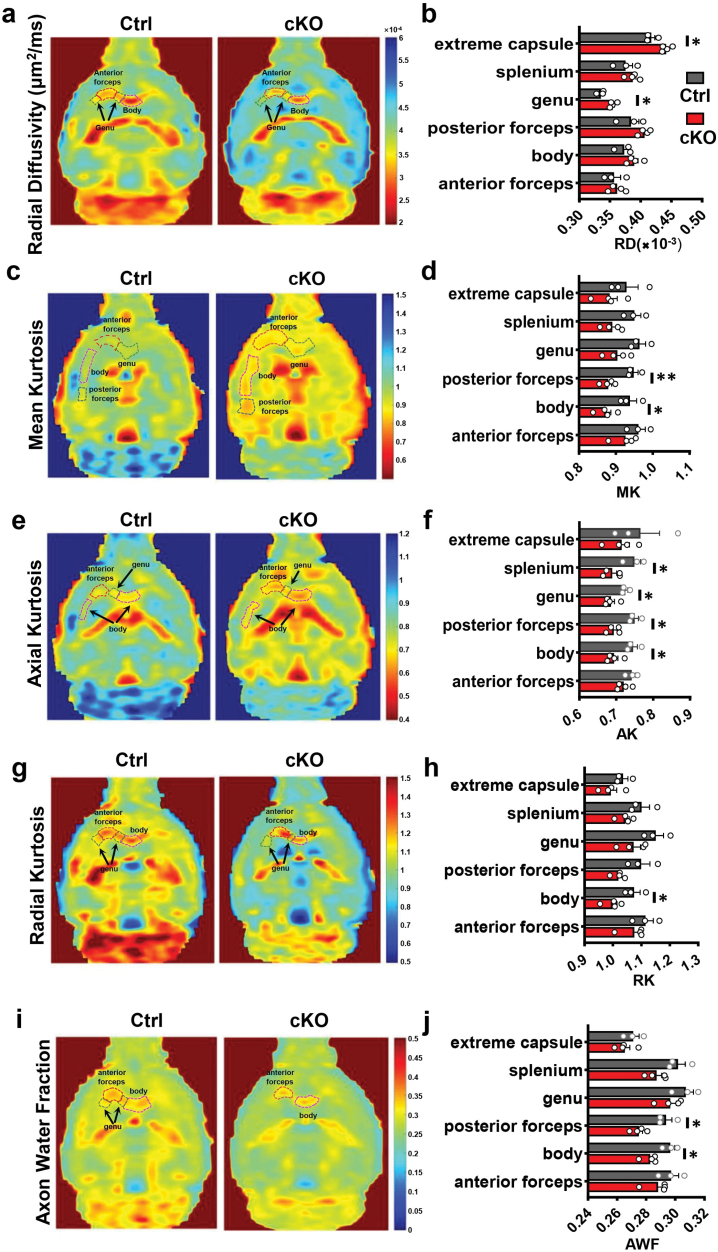
Representative DWI parameter maps and metrics between Ctrl and cKO mice. (a and b) Representative RD map and statistical results of different diffusion metrics in the region of interest (ROIs), including extreme capsule, splenium, genu, posterior forceps, body, and anterior forceps between Ctrl and *Mea6*-cKOs. (c and d) Representative MK map and statistical results. (e and f) Representative AK map and statistical results. (g and h) the representative RK map and statistical results. (i and j) Representative AWF map and statistical results. Ctrl: *n* = 3, cKO: *n* = 4 in all results about dMRI. Data represent the mean ± SEM. Data were analyzed via Student’s *t-*test. ^*^P < 0.05, ^**^P < 0.01.

We then adopted dMRI to inspect the microstructure of white matter, especially CC. Regions of interest were chosen based on the Allen mouse template (Supplementary Fig. S2a). We evaluated the sensitivity of different diffusion model derived metrics to detect hypomyelination in cKO mice. DTI derived metrics, mean diffusivity (MD) (Supplementary Fig. S2b), and radial diffusivity (RD) (Fig. 2a) showed significant differences between cKOs and controls. For the genu, both MD (Supplementary Fig. S2c) and RD (Fig. 2b) displayed a significant increase in cKOs. MD and RD also showed a significant increase for the posterior forceps and extreme capsule, respectively. DKI-derived metrics also showed significant changes between the two groups with different perspectives. Mean kurtosis (MK) decreases significantly in CC body and posterior forceps in cKOs (Fig. 2c and d). Axial kurtosis (AK) showed a significant decrease in the CC body, posterior forceps, genu, and splenium (Fig. 2e and f), and radial kurtosis (RK) [26] also showed a significant decrease in the CC body (Fig. 2g and h).

Both DTI- and DKI-derived metrics only provide an indirect measurement of microstructure of myelin sheaths by quantifying water diffusivity. The physical meaning of microstructure tissue parameters is still uncertain. Therefore, we introduced WMTI metrics, axonal water fraction [27, 28], derived from DKI to quantify hypomyelination in the mouse model with a new perspective. The axonal water fraction (AWF) also showed a significant decrease in body and posterior forceps (Fig. 2i and j). Moreover, significant hypomyelination of axons in the CC was confirmed in cKO group (manuscript in preparation). Together, these findings demonstrate that the microstructures of the CC are altered significantly in cKO mice.

### *Mea6* cKO results in reduced levels of cholesterols and triglycerides (TAGs), as well as total myelin sheath in the brain

**Figure 3:**
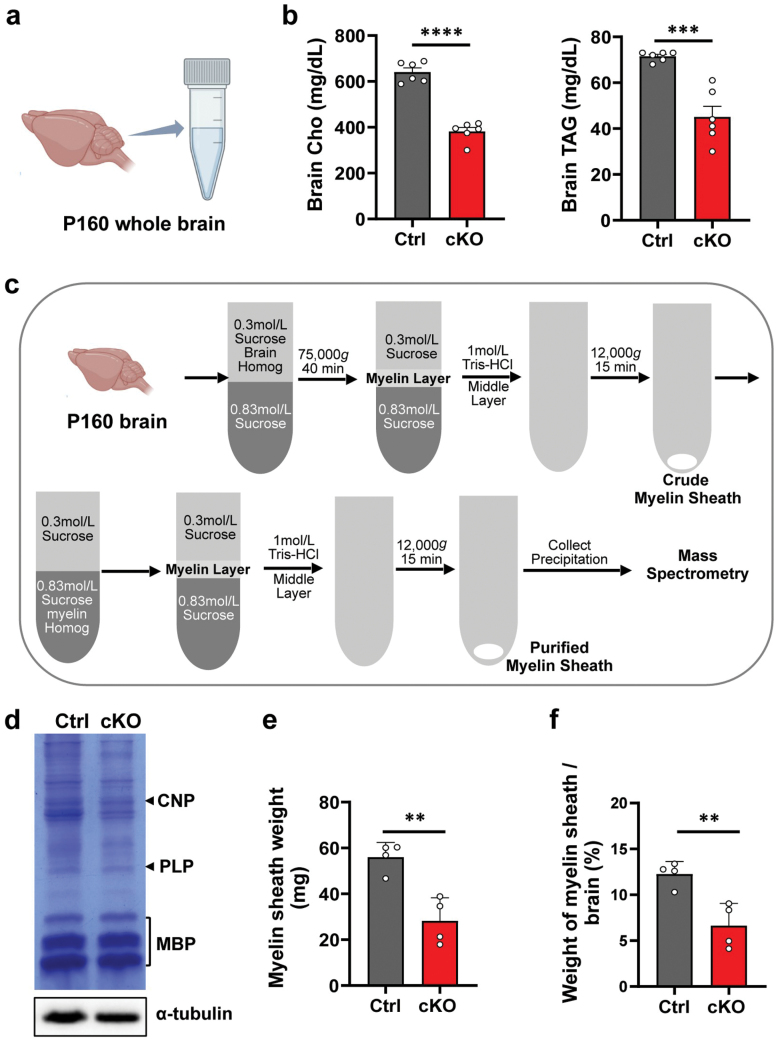
Decreased lipid contents and myelin sheath weight in *Mea6-cKO* brains. (a) Total lipids were extracted from whole brain homogenate. (b) Concentrations of cholesterol (Cho) and TAG in the *Mea6-cKO* brains are significantly reduced. Ctrl: *n* = 6, cKO: *n* = 6. (c) Schematic diagram of the isolation steps of myelin sheath from P160 mouse brains. (d) Coomassie blue staining of the purified myelin sheath. (e) Statistical results of myelin sheath weight in Ctrl and *cKO* groups. Ctrl: *n* = 4, cKO: *n* = 4. (f) Statistical results of percentage of myelin sheath weight to brain weight. Ctrl: *n* = 4, cKO: *n* = 4. The schematic diagrams of brain in (a) and (c) are prepared by BioRender. Data represent the mean ± SEM. Data were analyzed via Student’s *t-*test. ^**^P < 0.01, ^***^P < 0.001, ^****^P < 0.0001.

Based on the obvious abnormality of white matter, we postulated that *Mea6* cKO might result in the change of lipid contents. We extracted total lipids and analyzed the concentration of cholesterols and TAGs in whole brains (Fig. 3a). cKO brains exhibited significantly lower levels of cholesterols (382 ± 42.5 mg/dL vs. 641 ± 44.2 mg/dL) and TAGs (45 ± 11.6 mg/dL vs. 72 ± 2.1 mg/dL) than controls (Fig. 3b). As the most lipid-rich organ, myelin sheath accounts for around 30% dry weight of the human brain, and lipid from myelin sheath accounts for about 40% of total lipids in brain. Decreased cholesterol and TAG levels would reflect the change of myelin sheath in the brain, although TAGs are not membrane lipids. Therefore, we used the density gradient centrifugation to isolate myelin sheath [29] (Fig. 3c). Coomassie blue staining showed that myelin sheath was successfully isolated. Compared with myelin basic-protein (MBP), the majority of proteins in the purified myelin sheath of the *cKO* group were significantly reduced, including cyclic nucleotide phosphodiesterase (CNP) and myelin proteolipid protein (PLP) (Fig. 3d). In addition, the weight of myelin sheath (28.1 ± 10.1 mg vs. 55.9 ± 6.4 mg, Fig. 3e) and the ratio of myelin sheath weight to brain weight (6.6 ± 2.4% vs. 12.3 ± 1.4%, Fig. 3f) were obviously lower in cKO mice.

### Reduction of specific phosphatidylcholines in myelin sheath of cKO brain

**Figure 4:**
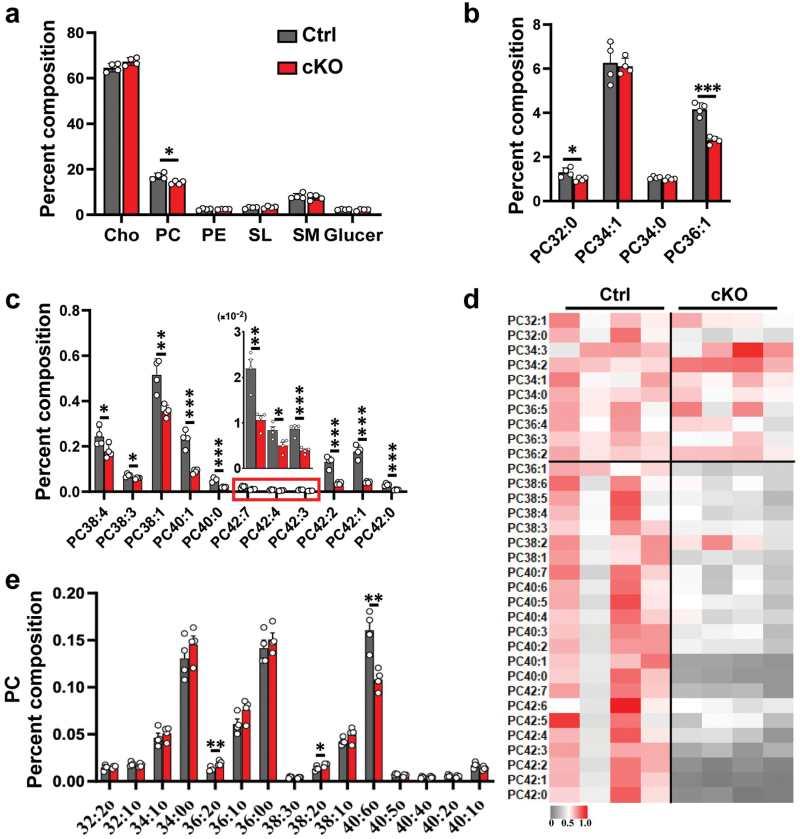
Significant reduction of specific PC in myelin from *Mea6-cKO* brains. (a) The content of PC in total lipids of myelin sheath is significantly reduced in cKO brains. Cho, cholesterol; PC, phosphatidylcholine; PE, phosphatidylethanolamine; SL, Sulfatides; SM, sphingomyelin; Glucer, Glucosylceramides. (b and c) The proportions of PCs with different carbon chain lengths in the total purified lipids. The result in the red boxes from B is enlarged and displayed directly above. (d) The heat map showed changing trend of the proportion of PC with different carbon chain lengths. (e) The proportion of modified PCs with different carbon chain lengths to the total purified lipids. Ctrl: *n* = 4, cKO: *n* = 4. Data represent the mean ± SEM. Data were analyzed via Student’s *t-*test. ^*^P < 0.05, ^**^P < 0.01, ^***^P < 0.001.

We next investigated whether white matter reduction would affect the lipid composition of myelin. Purified myelin sheaths were subjected to lipidomic analysis. A marked reduction in the proportion of phosphatidylcholine (PC) was detected in the myelin sheath of cKO mice (14.1 ± 0.8% vs. 16.9 ± 1.4%), while the levels of cholesterol, phosphatidylethanolamine (PE), sulfatides (SL), sphingomyelin (SM), and glucosylceramides (Glucer) were unchanged (Fig. 4a). Supplementation of PC, an important myelin lipid, can increase the number of myelinated segments of Schwann cells [30], suggesting that PC is important for myelin formation, either as a signal to induce differentiation or directly as a structural lipid. Therefore, PC reduction may contribute to leukodystrophy in our model mice.

Each lipid class contains individual lipids with different lengths of fatty acyl (FA) chain and varied degrees of unsaturation. The contribution of PC with different FAs to myelin structure and function is rarely reported. We thus analyzed the proportion of PC with different FAs in the myelin sheaths. We noticed that the proportions of various abundant PCs in total lipids in cKOs were significantly lower. Among the major PC species, PC36:1 was decreased by 33.7% (*P* = 0.0002) (Fig. 4b). Cuprizone administration led to the damage of CC and reduction of PC36:1 [31]. More importantly, PC36:1 level is high in white matter [32], but is significantly lower in the brains of multiple sclerosis patients with demyelination [31]. This result indicates that individual PC lipid can be used as potential biomarkers for myelin sheath degeneration or dysplasia.

The proportions of many other individual PCs were also significantly lower in cKO brains, and some lipids were significantly more prevalent, such as PC38:1, PC38:4, PC40:1, PC42:1, and PC42:2 (Fig. 4c). Moreover, we noticed an interesting phenomenon in the heat map for PC lipid species (Fig. 4d). The difference in PC lipid species between the two groups gradually increased with the growth of carbon chain, especially for PC42:0, PC42:1, and PC42:2 which were decreased by 74.4%, 76.2%, and 71.9% in cKOs, respectively (Fig. 4c and d). For most plasmalogen PC, the proportions were similar between the two groups, with only a few significant differences. For example, the proportions of PC36:2o and PC38:2o in cKOs increased by 33.4% (*P* = 0.009) and 23.1% (*P* = 0.035), respectively. However, the proportion of PC40:6o was decreased by 32.6% (*P* = 0.004) (Fig. 4e). These results denote a meaningful future direction for in-depth study of the function of PC lipid species in brain or myelin.

### Changes of other major myelin lipid composition in brains of *Mea6* cKO mice

**Figure 5:**
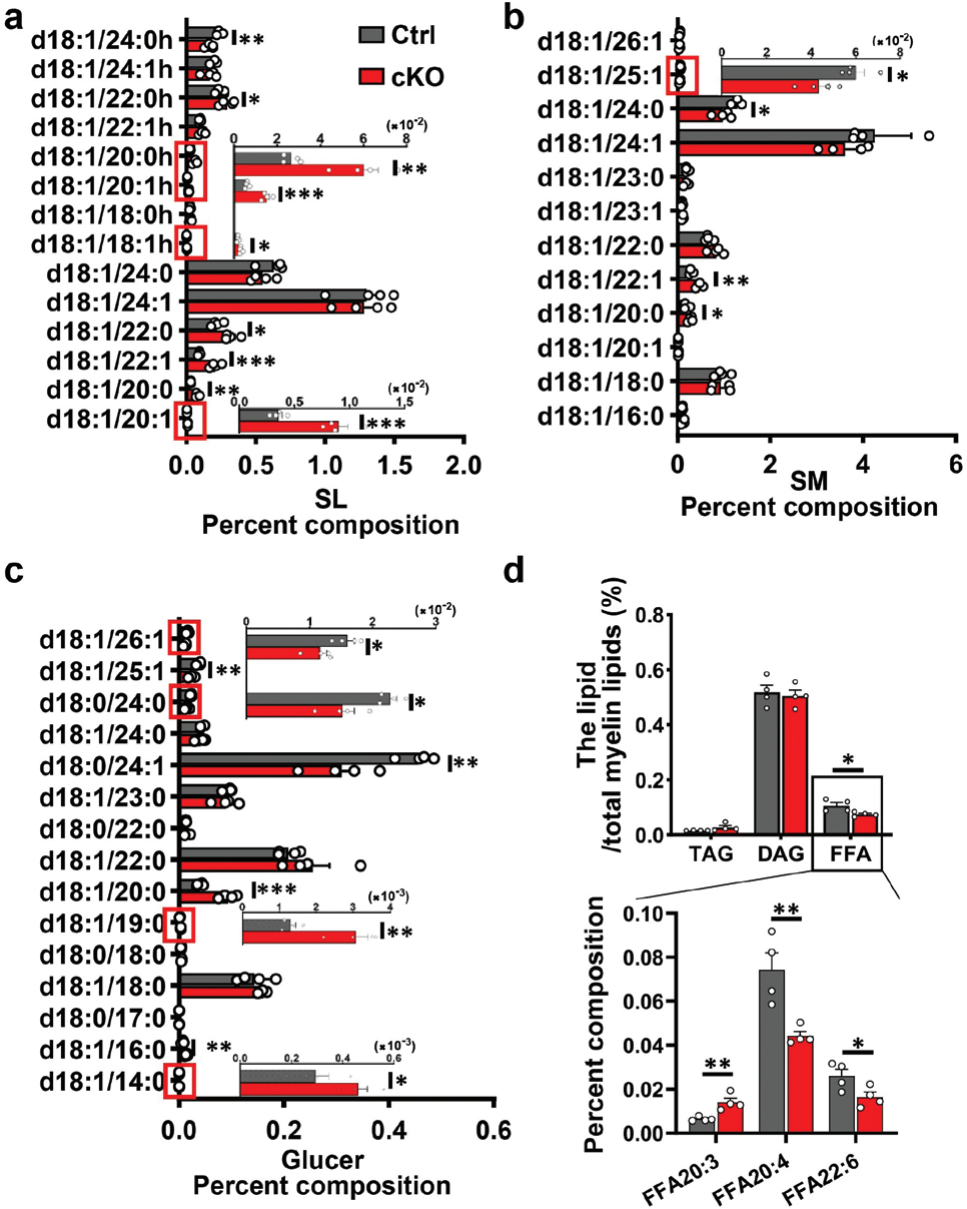
Changes of different myelin lipid composition in the myelin lipids from *Mea6-cKO* brains. The proportion of different lipids with different carbon chain lengths in total myelin lipids. (a) SLs. (b) Glucer. (c) SM. (d) The proportion of different non-membrane lipids in total lipids. SL, Sulfatides; SM, sphingomyelin; Glucer, Glucosylceramides; TAG, triacylglycerol; DAG, triacylglycerol; FFA, Free fatty acid. Ctrl: *n* = 4, cKO: *n* = 4. The results in the red boxes from (a), (b), and (c) are enlarged and displayed on the right. Data represent the mean ± SEM. Data were analyzed via Student’s *t-*test. ^*^P < 0.05, ^**^P < 0.01, ^***^P < 0.001.

We also inspected the proportion of other lipid species with different carbon numbers in total myelin lipids. Lipidomics revealed that total SL was comparable between the two groups (Fig. 4a). However, SL contains abundant individual lipids that were obviously different between the two groups (Fig. 5a). The ratios of most SL individual lipids with short-chain including SL20:1, SL20:0, SL22:0, SL22:1, SL18:1h (hydroxy), SL20:0h, and SL20:1h were significantly increased in cKOs, while SL24:0h, which belongs to VLCFAs, was significantly reduced (*P* = 0.009) (Fig. 5a). SM and Glucer showed a relatively consistent pattern: the proportions of different lipid species with a carbon number below 23 (C23) were increased in cKOs, while those VLCFAs (above C23) were significantly reduced (Fig. 5b and c). For example, SM20:0 and SM22:1 were significantly increased while SM24:0 and SM25:1 were significantly reduced (Fig. 5b), and Glucer14:0, Glucer16:0, Glucer19:0, and Glucer12:0 were obviously increased while Glucer24:1, Glucer24:0, Glucer25:1 and Glucer26:1 were significantly decreased (Fig. 5c). Combining the results of SL, SM, and Glucer, the proportions of most VLCFAs (carbon numbers above C23) in cKOs were reduced (Figs 4 and 5).

We also observed that some non-membrane lipids, such as free fatty acids (FFA), were reduced by 29.9% (*P* = 0.004) in cKOs (Fig. 5d). However, FFA of various carbon chains showed different changes, the ratio of FFA20:3 was increased by 54.2% in cKOs, while the ratios of FFA20:4 and FFA22:6 were decreased by 40.4% and 36.6% respectively (Fig. 5d). TAG and diacylglycerol (DAG) were similar between the two groups (Fig. 5d). We speculated that the reduction in total cholesterol and TAG content in the whole brain (Fig. 3b) might be caused by the defects in oligodendrocyte differentiation and reduced complexity, which in turn results in lower lipid requirements.

### *Mea6* cKO may affect the elongation of VLCFAs via affecting the expression of ELOVLs

**Figure 6:**
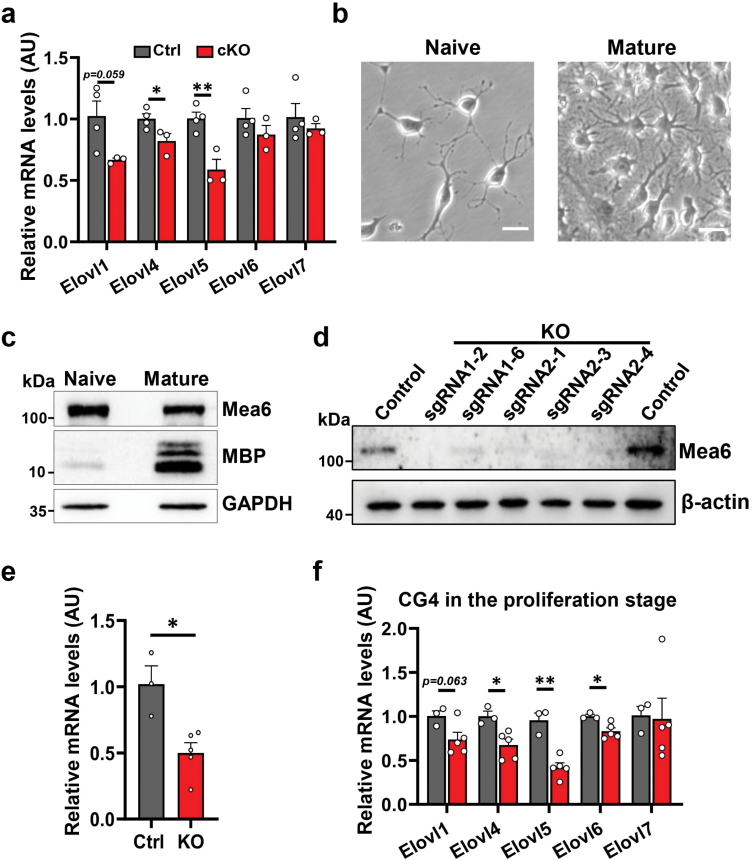
KO of *Mea6* results in reduced mRNA levels of *ELOVLs* both *in vivo* and *in vitro.* (a) Decreased expression of multiple *ELOVLs* in the CC of cKO brains. Ctrl: *n* = 4, cKO: *n* = 3. (b) Morphology of CG4 cells in the proliferative stage (naïve) and differentiated stage (mature). Scale bars: 20 μm. (c) Mea6 is expressed in both proliferative and differentiated CG4 cells. MBP: marker for mature CG4. (d) Western blotting shows knockout of *Mea6* in CG4 cells. The middle five samples are from independent monoclonal knockout lines. (e) The mRNA level of *Mea6* in KO cell line is significantly decreased. Ctrl: *n* = 3, KO: *n* = 5. (f) The CG4 shows decreased mRNA expression of multiple *ELOVLs* after KO of *Mea6*. Ctrl: *n* = 4, cKO: *n* = 3. Data represent the mean ± SEM. Data were analyzed via Student’s *t-*test. ^*^P < 0.05, ^**^P < 0.01.

Our above lipid profile results showed significant reduction of VLCFAs in cKOs in general. We, therefore, inspected whether the expression of elongase members, *ELOVLs* (*ELOVL1*–*7*), was affected in our model. According to the Tabula Muris database (single-cell transcriptomic data from *Mus musculus*), most *ELOVLs* are barely expressed in neurons, but show high abundance in glial cells (Supplementary Fig. S3). The mRNA levels of *ELOVL1*, *ELOVL5,* and *ELOVL6* are high in the entire oligodendrocyte lineage (Supplementary Fig. S3a, e and f), *ELOVL4* preferentially emerges at the precursor stage (Supplementary Fig. S3d), and *ELOVL7* appears predominantly in mature oligodendrocytes (Supplementary Fig. S3g). *ELOVL2* and *ELOVL3* are barely detectable in oligodendrocyte lines (Supplementary Fig. S3b and c). Therefore, we performed qPCR on the CC of P60 mice. The mRNA levels of *ELOVL4* (*P* = 0.047) and *ELOVL5* (*P* = 0.007) were significantly reduced in cKOs, and *ELOVL1* had a downward trend (*P* = 0.059) (Fig. 6a). *ELOVL6* and *ELOVL7* levels were similar between the two groups (Fig. 6a).

Most oligodendrocytes are mature in the CC of P60 mice and the various elongases are preferentially expressed in mature oligodendrocytes. Due to the tiny size of CC in neonatal mice, we decided to explore whether the changed mRNA levels were due to insufficient maturation of oligodendrocytes or impaired lipid chain elongation in rat oligodendrocyte cell line CG4 (Fig. 6b). After confirming the expression of *Mea6* in both proliferative (MBP^-^) and differentiated (MBP^+^) CG4 cells (Fig. 6c), we designed two sgRNAs and successfully knocked out *Mea6* using CRISPR Cas9 technology (Fig. 6d). Five monoclonal knockout (KO) strains with significantly reduced (*P* = 0.011) mRNA levels were obtained with the two sgRNAs (Fig. 6e).

We went on to inspect the mRNA levels of *ELOVLs* in the two groups and got very similar results as those in the CC (Fig. 6f). The mRNA levels of *ELOVL4* (*P* = 0.017) and *ELOVL5* (*P* = 0.001) in the *Mea6*-deletion group were significantly lower while the levels of *ELOVL1* showed a trend of decrease (*P* = 0.063) (Fig. 6f). The mRNA levels of *ELOVL7* were comparable between the two groups. Different from that in CC, the transcript level of *ELOVL6* (*P* = 0.018) was also significantly reduced in *Mea6*-KO CG4 cells (Fig. 6f). These results support the notion that elimination of *Mea6* affects the expression of ELOVLs and, in turn, the production of VLCFAs.

## Discussion

**Figure 7:**
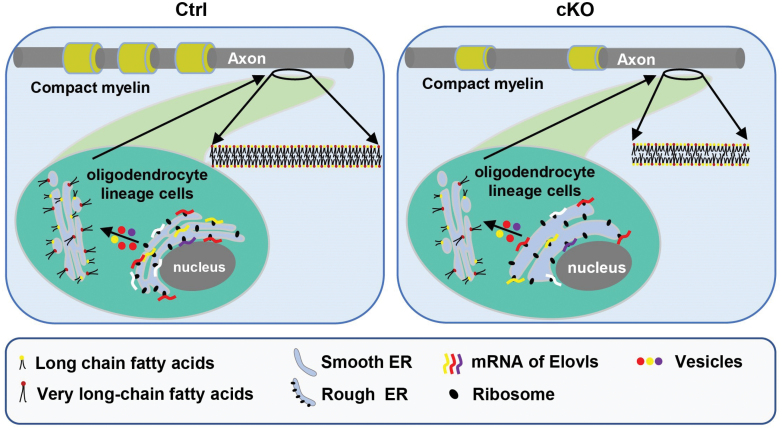
Model for the function of Mea6 in oligodendrocyte. Mea6 is involved in the transport of different lipids from the ER to the Golgi apparatus, and plays an essential role in the formation and/or maintenance of white matter. cKO of *Mea6* affects the composition of major myelin lipid, especially different VLCFAs−, leading to the hypomyelination of axons.

In this study, we show that Mea6 plays an essential role in the formation and/or maintenance of white matter, thus, confirming the hypothesis that members of COPII complex and Mea6 may be involved in oligodendrocyte and CC development [33, 34]. cKO of *Mea6* affects the composition of major myelin lipids, especially VLCFAs, leading to the hypomyelination of axons (Fig. 7).

Based on the histological finding of significant thinning of the CC in our *Mea6*^*fl/fl*^*;Olig2-Cre* mice, we combined different diffusion models of MRI to visualize and quantify the detailed microstructure changes of myelin sheath. Multiple parameters uncovered a global developmental deficit in the white matter of cKOs. For the DTI metrics, both MD and RD show a significant increase in several regions of CC, which indicates that water diffusion perpendicular to axons is easier in cKO brains [35]. In our study, increased RD may be the consequence of reduced layers of myelin sheaths or less lipid content in myelin which increases the permeability.

Similar to DTI metrics, RK provides a good estimation of hypomyelination in our cKO mice. It shows a significant decrease in the body of CC, which means that the diffusion displacement of water molecules deviates less from Gaussian distribution. This is consistent with the histological and DTI results, which confirms our hypothesis that Mea6 deletion-induced hypomyelination leads to thinner myelin sheaths and reduces lipid content in myelin. For other DKI metrics, both AK and MK metrics show similar results, which indicates fewer diffusion barriers and less structural complexity of the CC environment in cKO mice. We can hypothesize that the hypomyelination leads to not only reduced layers of myelin sheaths surrounding the axon but also reduced numbers of myelin sheaths along the axon. In addition, we also calculated the AWF index derived from WMTI, which was believed to be sensitive to axonal loss or demyelination. The AWF decreased significantly in both posterior forceps and body, which confirmed our assumption that *Mea6*-deletion-induced hypomyelination may alter the microstructure of CC by blocking myelin sheath formation. These different diffusion MRI methods can probe the morphological information of white matter microstructure from multiple perspectives, as well as myelin sheath deficit in *Mea6* cKO brains.

Considering the high abundance of lipid content in white matter, we analyzed and found that both cholesterol and TAG concentrations were significantly reduced in the whole brains of cKO mice. We then performed full lipidome profiling of purified myelin sheath. The most obvious change is the global reduction of PC with various chain lengths in cKOs. It would be intriguing to determine whether reduction of PC can be used as a biomarker for the diagnosis of white matter diseases. In addition, the proportions of VLCFAs between 24C and 26C in SL, SM, and Glucer were significantly reduced in cKOs. However, for those below 23C, all observed changes showed a consistent increase in cKOs.

VLCFAs were significantly reduced in general in cKOs, and this broad VLCFA alteration was very similar to that in *Elovl1* deletion mice [14]. Consistent with this, we found, both *in vivo* and *in vitro,* that knockout of *Mea6* resulted in decreased mRNA levels of multiple fatty acid elongases. The decrease of VLCFAs may explain some of the MRI parameters, such as AWF, which showed a significant reduction in the local CC in cKOs. Because VLCFAs have excellent water retention capacity, skin permeability barrier is disrupted in *Elvol4* mutant mice due to impaired synthesis of VLCFAs [36]. The reduction of VLCFA content may also cause changes in the microstructure and the fluid environment inside and outside the myelin sheath, thereby affecting the function of myelin sheath and axons. In addition to VLCFA insufficient, excessive accumulation of VLCFAs also causes dysmyelination and inflammatory-induced demyelination in some adrenoleukodystrophy (ALD)-patients [37]. These studies indicate that the appropriate content of various fatty acids is important for the stability of myelin sheath and the maintenance of its function. Although myelin sheath contains a lot of VLCFAs compared to other membranes [37], the exact function of VLCFAs in oligodendrocytes or myelin sheaths is still unclear.

After initiation of oligodendrocyte myelination, Mea6 is predicted to play an essential role in the transport of a large amount of lipids to the membrane. Why loss of Mea6 results in significant alterations in lipid species? The synthesis of short-chain fatty acids happens in the cytoplasm, while the elongation of VLCFAs occurs in the ER [38]. Mea6 is primarily localized on ERES and is also distributed throughout ER [34]. Therefore, Mea6 may be involved in the transport of VLCFAs or other structural lipids. It is possible that loss of Mea6 affects not only trafficking, but also the ER structure, the differentiation of oligodendrocytes and myelination, and thus the expression of ELOVLs. Reduction of total cholesterol or TAG and changes in myelin lipid composition in cKO brain may be the secondary effects due to defects in oligodendrocyte development. However, we cannot exclude the possibility that fatty acids play a role in oligodendrocyte differentiation, either as a structural component, supporting energy metabolism, or participating in signal transduction processes in our animal model [9, 39]. We are currently investigating whether Mea6 is essential for the differentiation of oligodendrocytes and the potential underlying mechanisms.

Taken together, Mea6 is essential for the development of white matter. The abnormal microstructure of the CC and hypomyelination could be confirmed by different diffusion MRI models. Our findings of the abnormal myelin lipid composition through full lipidome profiling will provide new insight into the pathogenesis of white matter diseases.

## Materials and methods

### Animals

*Mea6*^*Flox/Flox*^ mice and *Mea6* transgenic mice were previously generated [21–23]. Briefly, the flanking of the 11th exon in *Mea6* gene was inserted in two loxP sites. For *Mea6* transgenic mice, CAG promoter, LoxP-Stop-LoxP element (for conditional expression under the control of Cre), and *Mea6-GFP-KI* mice were constructed by our group and company. Briefly, we inserted the sequence around the last axon of *Mea6* in genome that can encode the EGFP protein.

### Perfusion and immunostaining analysis

Mice were anesthetized with ketamine (100 mg/kg) followed by transcardial perfusion with phosphate buffered saline (PBS) and 4% PFA. The dissected brains were fixed in 4% PFA overnight at 4°C, dehydrated in 30% sucrose, embedded in optimum cutting temperature [22] compound (Biopak Systems; Goleta, CA, USA), and sectioned (30-μm thickness) in 40-μm width slices. Cultured cells were fixed with PFA (4%) at room temperature for 15 min or with cold methanol at −20°C for 3 min. The brain slices or cultured cells were first incubated with blocking buffer (10% FBS, 5% BSA, 0.3% Triton X-100, 0.1% NaN_3_ dissolved in PBS) at room temperature for 1 h, then incubated in the primary antibody dissolved in the blocking buffer at 4°C for overnight or 37°C for 1 h, and finally incubated in the secondary antibody conjugated with Alexa Fluor 488, 568, or 647 dyes (1:2000) dissolved in the blocking buffer at room temperature for 1 h. The primary antibodies used for immunostaining included Rabbit-anti-Mea6/cTAGE5 (Sigma, Cat: HPA000387), Chicken-anti-GFP (Abcam, Cat: ab13970), Chicken-anti-MAP2 (Abcam, Cat: ab5392), Mouse-anti-Olig2 (Millipore, Cat: MABN50). Nuclei were counterstained with DAPI (4ʹ,6-diamidino-2-phenylindole) (Cell Signaling Technology, Cat: 4083s, 1:10000) when incubated with secondary antibody.

### Oligodendrocyte progenitor cell (OPC) isolation, cell cultures, and CRISPR Cas9 technology

The OPCs were isolated from P0 to P3-d-old wild type and cKO pups as previously described. Briefly, cortex from P0 to P3 mice was dissected and dissociated with accutase for 13 min at 37°C, blown into single-cell suspension, and then seeded onto the poly-d-lysine coated coverslips in proliferation medium (DMEM/F12 supplemented with 2% B-27 supplement, 1% N-2, 10 ng/mL EGF, 10 ng/mL PDGFRα, and 1% GlutaMAX). The culture dish must be shaken to suspend the cell debris the next day, and then replaced with fresh culture medium. After that, the medium should be half replaced with fresh medium every 2 days. When the cells reached the proper density, the medium was changed to the DMEM supplemented with 2% B-27 supplement, 5 μg/mL insulin, 1% GlutaMAX, 10 mg/mL Holo-transferrin, 0.5% FBS, 50 ng/mL CNTF, and 1% 100 × OL-supplement for the differentiation. 100 × OL-supplement: 1.02 g BSA, 0.6 mg progesterone, 161 mg putrescine, 0.05 mg sodium selenite, and 4 mg T3 dissolved into 100 mL DMEM. Oligodendrocytes were identified by immunofluorescence staining for lineage marker Olig2 and the purity reaching more than 90%.

To generate CG4 cell lines in which Mea6 expression was abolished, we chose the CRISPR/Cas9 system. Briefly, we designed sgRNAs in the second axon of *Mea6* through the CRISPOR-TEFOR (tefor.net/portfolio/crispor) and inserted them in the PX458 plasmid. After 24 h transfection with Lipo3000, GFP positive CG4 cells were isolated by fluorescence-activated cell sorting (FACS), and single cells were seeded onto poly-d-lysine coated 96-well plates. Medium conditions were consistent with those of primary OPCs. Sequencing and western blot were used to verify KO efficiency. All cells were cultured at 37°C with 5% CO_2_.

The following sgRNA sequences were used for eliminating of *Mea6* in CG4:

sgRNA1: agagcagacttcaatcccaa; sgRNA2: gcgatatgtgtgcttaccgt

### Western blotting and gray statistics

Brain tissues from all groups of mice and cells were lysate in RIPA as previous described [23]. The protein concentration was measured by the Bio-Rad method. Samples of 10–20 μg of protein were subjected to different concentrations of SDS–polyacrylamide gel electrophoresis (SDS–PAGE). All the western blotting was representative of at least three individual experiments. Expression levels of all proteins were evaluated by gray value with Image J, and the average levels of relative abundance of each protein in control mice or cells were normalized to 1. The primary antibodies used for immunoblotting were Rabbit-anti-Mea6/cTAGE5 (Sigma, Cat: HPA000387), Rat-anti-MBP (Abcam, Cat: ab7349), Mouse-anti-α-tubulin (Cell Signaling Technology, Cat: 3873s), Mouse-anti-GAPDH (Cell Signaling Technology, Cat: 97166s), and Mouse-anti-β-actin (Cell Signaling Technology, Cat: 3700s).

### MRI and analysis

*In vivo* MRI experiments were conducted on a 11.7T Bruker scanner (Bruker Biospec, Paravision360, Germany). Mice were anesthetized using an isoflurane vaporizer with the following concentrations, 3% for the induction, and 1%–1.5% during data acquisition. Body temperature was maintained at 37.0 ± 0.2°C using heated water, and the four-channel surface coil was used for the RF reception.

TurboRARE sequence for Axial T_2_-weighted images were obtained with TR = 6500 ms, effective TE = 30 ms, FOV = 19 × 11 mm^2^, matrix = 320 × 192, spatial resolution = 0.06 × 0.06 mm^2^, slice thickness = 0.24 mm, total scan time = 9.7 min. DW-EPI sequence for multi-shell diffusion weighted imaging, 2 non-diffusion weighted images and 120 diffusion weighted images were acquired. The sequence parameters were TR/TE = 3500/18.8 ms,δ/∆ = 3.5/9.24 ms, slice thickness = 0.3 mm, FOV = 18 mm × 12 mm, spatial resolution = 0.3 × 0.3 mm^2^, flip-angle = 90°, 4 segments, 60 non-collinear diffusion gradient directions and two *b*-values for each gradient direction (1000, 2500 s/mm^2^), total scan time = 28.5 min.

All image data transferred into Digital Imaging and Communications in Medicine (DICOM) format by MRIcron. Manually mouse skull stripping based on T_2_-weighted image and b0 image in diffusion MRI by ITK-SNAP [40]. The quantitative indices of different diffusion models were derived using FSL [41] and DESIGNER [42].

### Total lipid extraction and detection in brain

The brains of 4-month-old mice were quickly dissected on ice and weighed. 5% Triton X-100 prepared from PBS was proportionally added. For example, 2 µL 5% Triton X-100 was added to a 1 µg sample. The tissue was homogenized in the tissue crusher for 30 s, forming a homogenate state. The brain lysate was incubated in an 80°C water bath for 10 min, taken out, and cooled to room temperature. Repeated steps of incubation and cooling led to adequate extraction of lipids. The homogenate was centrifuged for 10 min at 12,000 *g*, and the supernatant was collected, which can be stored in the refrigerator at −80°C for a long time. The prepared samples were sent to the Animal Testing Center of China Agricultural University to detect the concentration of cholesterol and TAGs.

### Myelin purification

Isolation of myelin referred to previous reports [29]. It mainly used the characteristic of different density of myelin to other components. Briefly, fresh adult brain was homogenized in 0.3 mol/L sucrose solution containing 20 mmol/L Tris-HCl buffer (pH 7.45), 1 mmol/L EDTA and 1 mmol/L DTT, 100 µmol/L phenylmethylsulfonylfluoride (PMSF) (these compounds must be presented in all steps of the procedure). The homogenate was layered over 0.83 mol/L sucrose and centrifuged 35 min at high speed (75,000 *g*). The band of crude myelin membranes occurring at the 0.3 mol/L and 0.83 mol/L sucrose interface was collected, and after washing and resuspend by 20 mL Tris-HCl, this myelin fraction was centrifugated with speed 12,000 *g* for 15 min. The collected myelin was resuspended in 0.83 mol/L sucrose again, and the 0.83 mol/L sucrose solution overlaid with 0.30 mol/L sucrose. After repeated centrifugation, myelin floats band located at the 0.30 mol/L and 0.83 mol/L sucrose interface. Collected myelin band and centrifuged the myelin suspension 10 min at 12,000 *g*, 4°C, we got the purified myelin for lipid mass spectrum after discarding the supernatant. Samples not used temporarily can be frozen to −80°C.

### Fine extraction and processing of myelin lipids

Lipids were extracted using the improved Bligh and Dye extraction method [43]. Mouse myelin samples were collected in 2 mL Sarstedt centrifuge tubes. Nine hundred μL of ice-cold chloroform: methanol (1:2) with 10% water was added to inactivate the myelin sheath. Larger tissues were then minced with clean scissors. The samples were pre-cooled at −20°C for 30 min. Two scoops of clean ceramic beads were added to the samples and homogenized in a bead mill (OMNI, Kennesaw, GA) using an optimized program. The samples were incubated for 30 min at 1500 rpm on a 4°C shaker. After a quick centrifugation, 350 μL MilliQ water and 300 μL chloroform were added and vortexed to mix. After centrifugation at 12,000 rpm for 5 min, the lower organic phase was extracted into a new 1.5 mL centrifuge tube. Five hundred μL chloroform was added to the remaining aqueous phase, vortexed, incubated on a 4°C shaker at 1500 rpm for 10 min, and centrifuged at 12,000 rpm for 5 min, and the lower organic phase was extracted into the same 1.5 mL centrifuge tube as in the previous step. All organic phases collected were evaporated to dryness in Speed Vac in OH mode. Analytical instrument model was AB SCIEX JasperTM HPLC-Triple Quad 4500MD and Agilent 1260-AB SCIEX QTrap 5500.

The analyses of phospholipids and sphingolipids were carried out on the system of AB SCIEX JasperTM HPLC-Triple Quad 4500MD. The lipid extract was separated using Phenomenex Luna Silica 3mm chromatographic column (inner diameter 150 mm × 2.0 mm), and chromatographic conditions were set as mobile phase A (Chloroform:methanol:ammonium hydroxide, 89.5:10:0.5) and mobile phase B (chloroform:ethanol:ammonium hydroxide:water, 27:65:0.5:7.5). Quantitation was calibrated using an internal standard. Internal standard mixtures included DMPC, DMPE, DMPG, d31-PS, diC8-PI, C17-PA, C14-LBPA, C17-LPC, C17-LPA, C17-LPS, C17-LPI, C17-LPG, Cardiolipin MIX (CL86:4, CL80:4, CL61:1; CL57:4), C12-SM, C15 Ceramide-d7 (d18:1-d7/15:0), C8-GluCer, C8-GalCer, d3-LacCer d18: 1/16:0, GM3-d18:1/18:0-d3, d17:1-S1P, d17:1-Sph, C17 Gb3, C17-SL, d3-16:0-carnitine, FFA-d31-16:0, and FFA-d8-20:4.

Glyceride omics analysis was performed on Agilent 1260-AB SCIEX QTrap 5500 LC-MS system [44]. The separation of TAGs and DAGs was performed on a Phenomenex Kinetex C18 2.6 mm chromatographic column (inner diameter 4.6 mm × 100 mm) using an Equi Gradient mobile phase containing chloroform:methanol:0.1 mol/L ammonium acetate 100:100:4 (v/v/v), with a flow rate of 300 μL/min, lasting for 10 min. The content of short-chain, medium-chain, and long-chain TAG is calculated by referring to the internal standards of TAG (14:0) 3-d5, TAG (16:0) 3-d5, and TAG (18:0) 3-d 5. DAG-d5 (1,3-17:0) and d5-DAG1 8:1/18:1 were used as internal standards to quantify DAG.

Quantitative analysis of cholesterol and cholesterol ester was carried out on Agilent 1260-AB SCIEX QTrap 5500 [45]. It was separated on Agilent Eclipse XDB-C18 column (inner diameter 4.6 mm × 150 mm), using an equal gradient mobile phase containing chloroform:methanol 100:100 (v/v), with a flow rate of 650 μL/min, lasting for 6 min. d6 CE and d6 Cho were used as internal standards for quantification.

### RNA extraction and real-time quantitative PCR

Total RNA was extracted from brain tissue and cultured cells using TRIZOL reagent (Invitrogen). Briefly, 1 mL TRIZOL was used to dissolve the brain tissues or cultured cells and 200 μL chloroform was subsequently added. After precipitation with isopropanol and washed with 70% (vol/vol) cold ethanol, the mRNA was collected and reversely transcribed into cDNA using the reverse transcriptase (Promega). A 2 X SYBR Green PCR mixture (Bio-Rad) was used to perform real-time PCR and analyzed with Bio-Rad C1000 Thermal Cycler. The following primer sequences were used for the mouse genes:

*Actin* forward, agggaaatcgtgcgtgac; reverse, gatagtgatgacctgaccgt;

*Elovl1* forward, ggcctcgaatcatggctaat; reverse, cagatgaggtggatgatgatgg;

*Elovl4* forward, gacctggaccattgcagataa; reverse, ggtatcgcttccaccaaagata;

*Elovl5* forward, cccgagatacaagagtcaaagg; reverse, gtcccagccatacaatgagtaa;

*Elovl6* forward, gctgtacgctgcctttatct, reverse, cttcgtggctttcttcactttg;

*Elovl7* forward, tcctgggcctctatgtctatt; reverse, cgttatcatcgctttcttgagttc;

The following primer sequences were used for the rat genes:

*Gapdh* forward, actcccattcttccacctttg; reverse, ccctgttgctgtagccatatt;

*Elovl1* forward, ccctacctttggtggaagaaa; reverse, ccagatgaggtggatgatgatag;

*Elovl4* forward, gacctggtccattgcagataa; reverse, gccacacgaacaggagatag;

*Elovl5* forward, ggtgtgtgggaaggcaaata; reverse, ccaccagaggacacgaataac;

*Elovl6* forward, gctgatgggctgtgtcatta; reverse, catgagtgaggaccagaagatg;

*Elovl7* forward, gactattctcagtcgccaagag; reverse, agatagtgtcaaacagctcgataa;

*Mea6* forward, ttggacatgaagagtggcctaga; reverse, aagcgccctttggctgat;

## Supplementary Material

load010_suppl_Supplementary_Material

## Data Availability

All study data are included in the article and/or supporting information.
