# Supplementary material for: Ablation of Mea6/cTAGE5 in oligodendrocytes significantly impairs white matter structure and lipid content
Source: Life Metab. 2023 Mar 23;2(2):load010. doi: 10.1093/lifemeta/load010 (PMC11748983; doi:10.1093/lifemeta/load010)
Supplement: load010_suppl_Supplementary_Material [file load010_suppl_Supplementary_Material.docx]

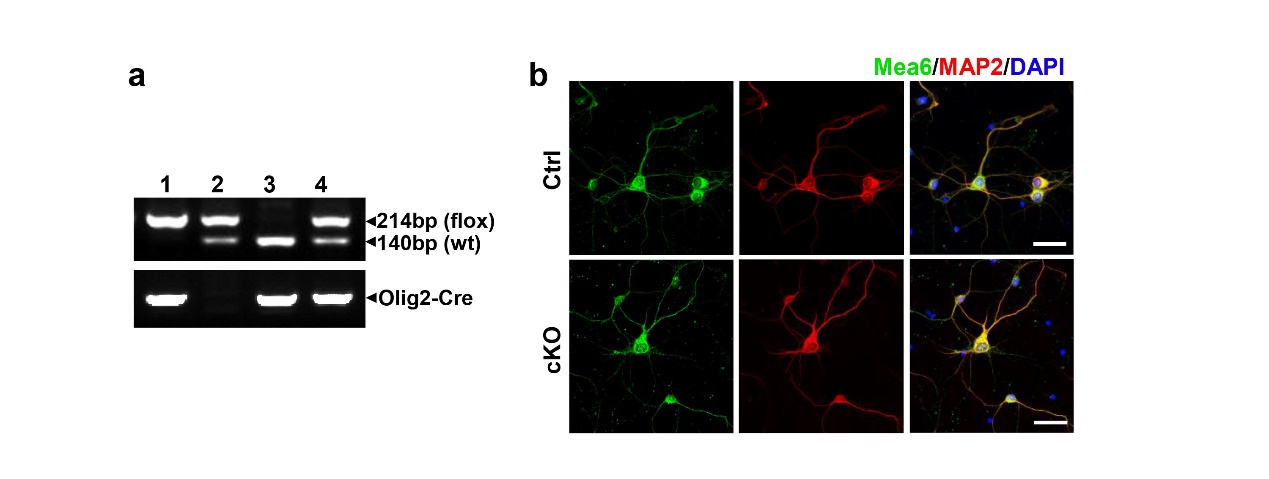


**Figure S1: Mea6 is expressed normally in neurons of *Mea6-cKO* mice**

(a) PCR genotyping of littermates. Lane 1: *Mea6^flox/flox^; Olig2-Cre* (cKO); Lane 2: *Mea6^flox/+^* (Ctrl); Lane 3: *Mea6^+/+^; Olig2-Cre* (Ctrl); Lane 4: *Mea6^flox/+^; Olig2-Cre* (heterozygous). (b) Expression of Mea6 in cultured neurons from WT and *Mea6-cKO* mice. Scale bars: 40 μm.


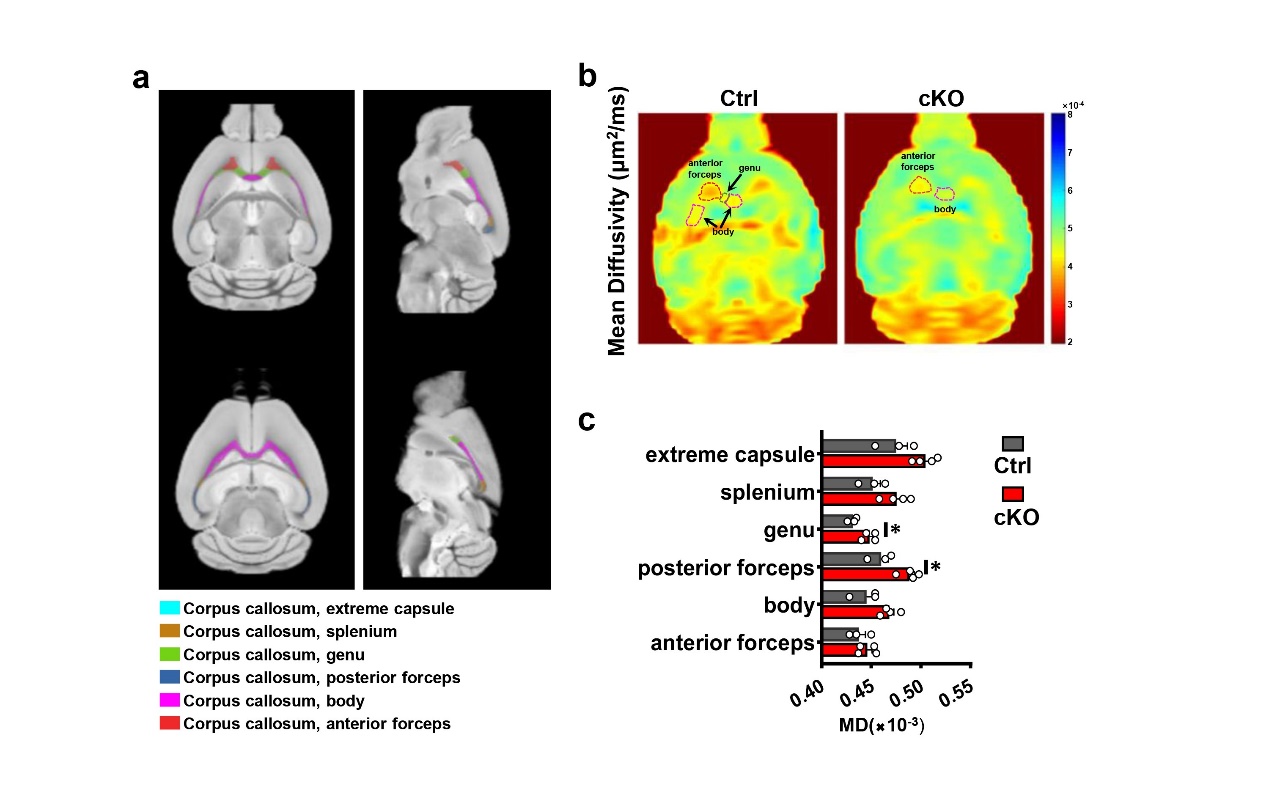


**Figure S2: Representative DWI parameter maps and metrics.**

(a) Region of interest chosen based on the Allen mouse brain map. (b) and (c) are the representative MD map and statistical results between the two groups. (Ctrl：n = 3; cKO：n = 4). Data represent the mean± SEM. **P* < 0.05. Student’s *t*-test.


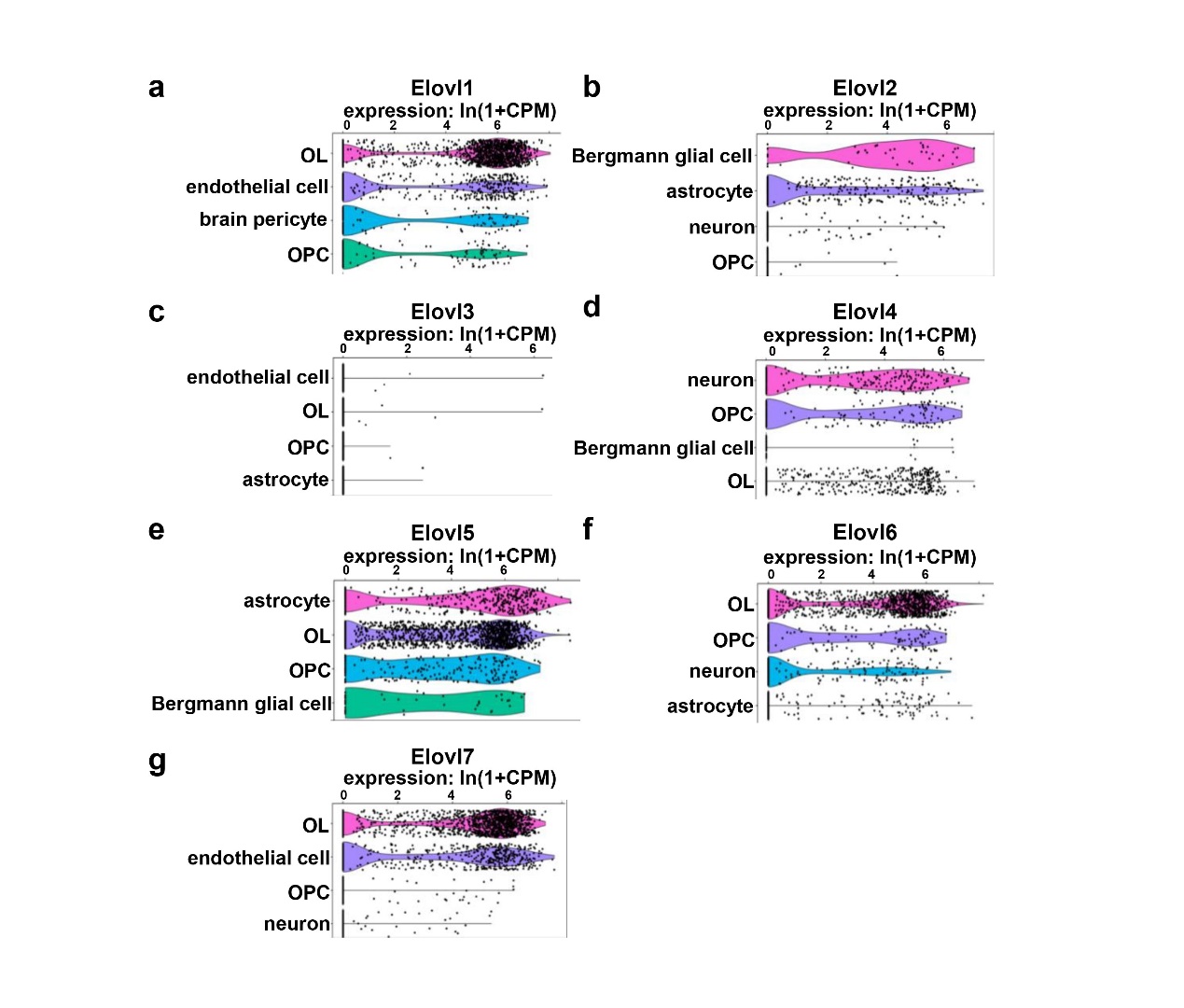


**Figure S3: mRNA levels of the ELOVL family in different cell types according to the Tabula Muris database (single-cell transcriptomic data from Mus musculus).**

(a) ELOVL1 is highly expressed in oligodendrocytes. (b) ELOVL2 is barely expressed in neurons and OLs. (c) ELOVL3 is barely expressed in the nervous system. (d) ELOVL4 is mainly expressed in neurons and OPCs. (e) ELOVL5 is mainly expressed in glial cells. (f) ELOVL6 is mainly expressed in the OL lineage. (g) ELOVL7 is mainly expressed in OLs and endothelial cells.
